# *O*-(2-[^18^F]fluoroethyl)-l-tyrosine PET in gliomas: influence of data processing in different centres

**DOI:** 10.1186/s13550-017-0316-x

**Published:** 2017-08-16

**Authors:** Christian P. Filss, Nathalie L. Albert, Guido Böning, Elena Rota Kops, Bogdana Suchorska, Gabriele Stoffels, Norbert Galldiks, Nadim J. Shah, Felix M. Mottaghy, Peter Bartenstein, Jörg C. Tonn, Karl-Josef Langen

**Affiliations:** 1 Institute of Neuroscience and Medicine (INM-4, INM-3), Forschungszentrum Jülich, Jülich, Germany; 2 Department of Nuclear Medicine, RWTH University of Aachen, Aachen, Germany; 3 Department of Nuclear Medicine, LMU Munich, Munich, Germany; 4 Department of Neurosurgery, LMU Munich, Munich, Germany; 5 Department of Neurology, University of Cologne, Cologne, Germany; 6 Center of Integrated Oncology (CIO), Universities of Cologne and Bonn, Bonn, Germany; 7 Department of Neurology, RWTH University of Aachen, Aachen, Germany; 8 Jülich-Aachen Research Alliance (JARA) - Section JARA-Brain, Jülich and Aachen, Germany

**Keywords:** Brain tumours, FET PET, Tumour-to-brain ratios, Dynamic FET PET

## Abstract

**Background:**

PET using *O*-(2-[^18^F]fluoroethyl)-l-tyrosine (^18^F-FET) is an established method for brain tumour diagnostics, but data processing varies in different centres. This study analyses the influence of methodological differences between two centres for tumour characterization with ^18^F-FET PET using the same PET scanner.

Methodological differences between centres A and B in the evaluation of ^18^F-FET PET data were identified for (1) framing of PET dynamic data, (2) data reconstruction, (3) cut-off values for tumour delineation to determine tumour-to-brain ratios (TBR) and tumour volume (T_vol_) and (4) ROI definition to determine time activity curves (TACs) in the tumour. Based on the ^18^F-FET PET data of 40 patients with untreated cerebral gliomas (20 WHO grade II, 10 WHO grade III, 10 WHO grade IV), the effect of different data processing in the two centres on TBR_mean_, TBR_max_, T_vol_, time-to-peak (TTP) and slope of the TAC was compared. Further, the effect on tumour grading was evaluated by ROC analysis.

**Results:**

Significant differences between centres A and B were found especially for TBR_max_ (2.84 ± 0.99 versus 3.34 ± 1.13; *p* < 0.001), T_vol_ (1.14 ± 1.28 versus 1.51 ± 1.44; *p* < 0.001) and TTP (22.4 ± 8.3 min versus 30.8 ± 6.3 min; *p* < 0.001) and minor differences for TBR_mean_ and slope. Tumour grading was not influenced by different data processing.

**Conclusions:**

Variable data processing of ^18^F-FET PET in different centres leads to significant differences especially for TBR_max_ and T_vol_. A standardization of data processing and evaluation is needed to make ^18^F-FET PET comparable between different centres.

## Background

PET using the amino acid *O*-(2-^18^F-fluoroethyl)-l-tyrosine (^18^F-FET) has received increasing attention for brain tumour diagnostics due to logistic advantages of ^18^F labelling (half-life, 109.8 min) compared with l-[methyl-^11^C]-methionine (^11^C-MET) PET, efficient radiosynthesis and high in-vivo stability [1–6]. Multiple studies have demonstrated the clinical potential of ^18^F-FET PET to determine the extent of cerebral gliomas for biopsy guidance, treatment planning, detection of tumour recurrence, estimation of prognosis in newly diagnosed and untreated gliomas and treatment monitoring [7–12]. ^18^F-FET uptake in the tumour is usually expressed by mean and maximum tumour-to-brain ratios (TBR_mean_, TBR_max_) within a scan period between 20 and 40 min after injection. Furthermore, the time-activity curves (TACs) of ^18^F-FET uptake typically show differences in high-grade and low-grade gliomas or nonneoplastic lesions which provide valuable additional information for tumour grading or differential diagnosis [13–15]. Thus, continuously increasing ^18^F-FET uptake is more frequently observed in low-grade gliomas and nonneoplastic lesions, while kinetics with an early peak of ^18^F-FET uptake in the first 10–20 min after injection followed by a decreasing TAC is a common finding in more aggressive tumours like high-grade glioma or brain metastases [10, 16, 17]. TBR and dynamic parameters of ^18^F-FET uptake may be influenced on the one hand by the spatial resolution of the PET scans which is dependent on the scanner type, reconstruction algorithms and data filtering and on the other hand by the definition of the region of interest (ROI) in the tumour and the brain. There is some controversy about the diagnostic accuracy of TAC analysis for tumour grading raising the question whether discrepant results in different centres may be caused by differences in the methodology, patient population or other reasons such as neuropathological interpretation.

In the present study, methodological differences between two large centres in Germany (A = Ludwig-Maximilians-University Munich, Germany; B = Forschungszentrum Jülich, Germany) with a high number of ^18^F-FET PET investigations (> 500/year) and multiple publications in the field were identified. Based on a balanced group of 20 patients with high-grade glioma of WHO grade III and IV (HGG) and 20 patients with low-grade glioma of WHO grade II (LGG) [18] who were investigated with the same scanner used in both centres, the influence of methodological differences between the two centres on common parameters for tumour characterization with ^18^F-FET PET was evaluated. This analysis may be helpful to develop a more standardized approach in order to make the data between different centres more comparable.

## Methods

### Patient population

The data of 40 adults, previously untreated patients with brain tumours who were investigated with ^18^F-FET PET at the Forschungszentrum Jülich between June 2006 and April 2014, were selected randomly from our data base and included in this study. Histopathological diagnosis was available for all patients obtained either by biopsy or tumour resection which was performed within 3 months after ^18^F-FET PET. Diagnosis was astrocytoma WHO grade II in 20 cases, anaplastic astrocytoma WHO grade III in 10 cases and glioblastoma WHO grade IV in 10 cases according to Louis et al. [18]. All patients were investigated within a prospective study evaluating the diagnostic value ^18^F-FET PET in cerebral gliomas which was approved by the university ethics committee and federal authorities (study no. 2438, University of Düsseldorf). All subjects gave prior written informed consent for their participation in the ^18^F-FET PET study and evaluation of their data for scientific purposes.

### ^18^F-FET PET

The amino acid ^18^F-FET was produced via aminopolyether-activated nucleophilic ^18^F-fluorination and applied as described previously [19]. Dynamic PET data were acquired in list mode for 40 min after intravenous injection of approx. 200 MBq ^18^F-FET. The measurements were performed on an ECAT Exact HR+ scanner in 3D mode (Siemens Medical Systems, Knoxville, TN, USA: 32 rings, axial field of view 15.5 cm, image resolution 5.5 mm) which is routinely used for ^18^F-FET PET in both centres. For attenuation correction, transmission scans with three ^68^Ge/^68^Ga rotating line sources were measured. Raw PET data were corrected for random and scattered coincidences as well as dead time.

### Data processing

**Fig. 1:**
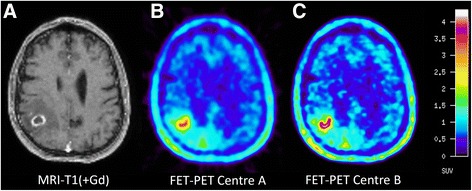
Glioblastoma in the right parietal lobe. Contrast-enhanced T1-weighted MRI (**a**) shows a ring-enhancing lesion. FET PET image based on the method of centre A (**b**) shows lower noise but the image based on the method of centre B (**c**) shows a sharper demarcation of the metabolically active tumour parts

**Fig. 2:**
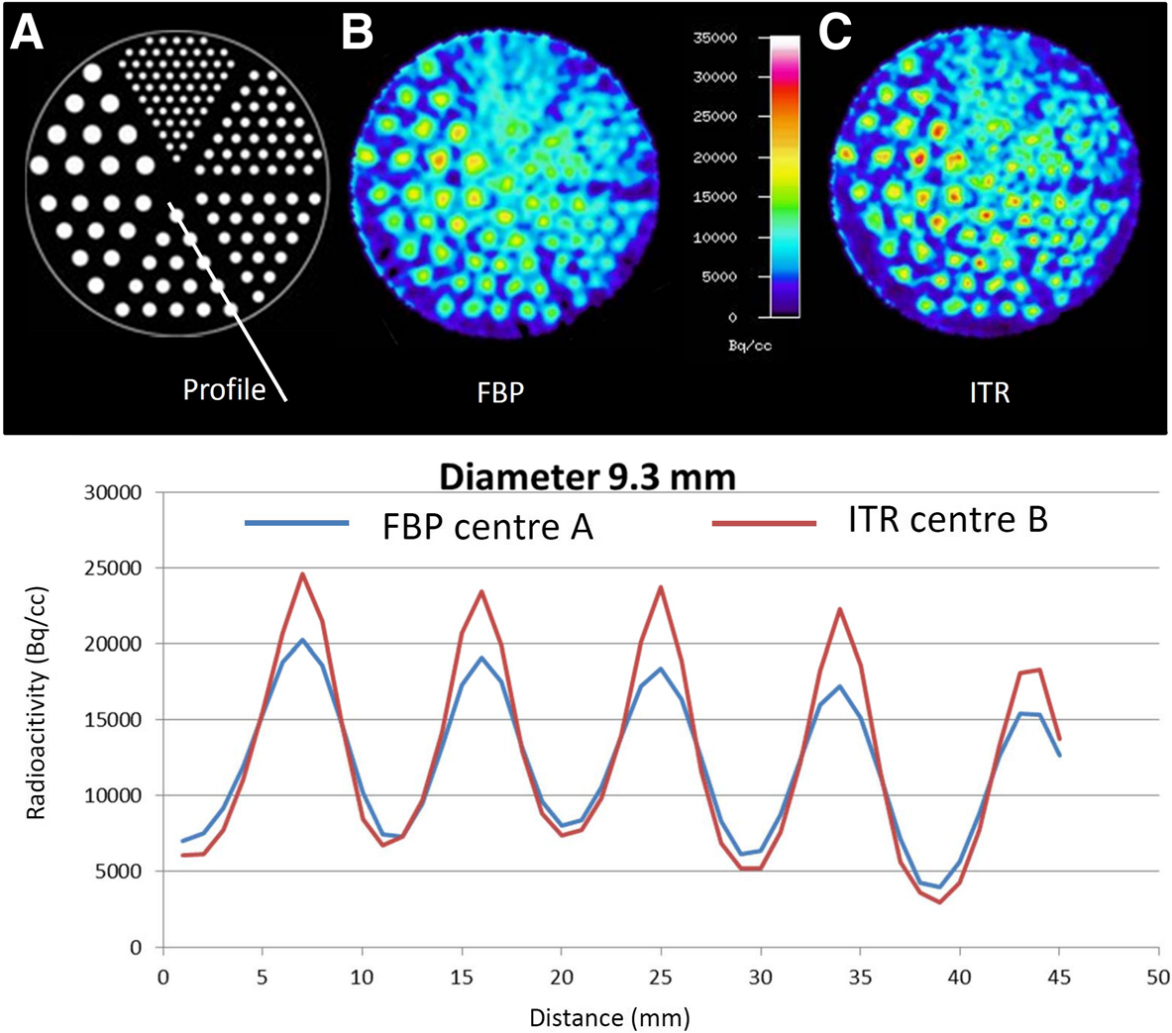
Jasczack phantom (**a**) with tubes of different size filled with radioactivity and reconstructed according to the procedure in centre **A** (**b**, blue profile line) and centre **B** (**c**, red profile line). The method of centre **A** shows about 20% lower maximum values in the tubes with a diameter of 9.3 mm which is mainly due to reconstruction by filtered back projection in centre **A** instead of iterative reconstruction (centre **B**)

Methodological differences between centres A and B were identified for the framing of dynamic PET data and data reconstruction (Table 1). Therefore, the PET data were processed in two different ways according to the individual approach established in centre A and centre B. For centre A, the dynamic data set was framed into time intervals of 6 × 10 s, 4 × 30 s, 1 × 2 min, 3 × 5 min and 2 × 10 min and the data were reconstructed by filtered back projection (FBP) using a 5-mm Hann filter. For centre B, the framing was 5 × 1 min, 5 × 3 min and 4 × 5 min followed by iterative reconstruction (ITR) (ordered-subset expectation maximization, 6 iterations, 16 subsets). ^18^F-FET uptake in the tissue was expressed as standardized uptake value (SUV) by dividing the radioactivity (kBq/ml) in the tissue by the radioactivity injected per gram of body weight. An example of a brain tumour for the time interval from 20 to 40 min after injection for the different data processing in centres A and B is shown in Fig. 1. Corresponding evaluation of a Jaszczak phantom with the two methods is shown in Fig. 2.

**Table 1:** Comparison of methodology centre A and centre B

	Centre A	Centre B
	LMU	FZJ
Scanner	ECAT Exact HR+ PET	ECAT Exact HR+ PET
Framing	6 × 10”, 4 × 30”, 1 × 2’, 3 × 5’, 2 × 10’	5 × 1’, 5 × 3’, 6 × 5’
Reconstruction	FBP (5-mm Hann filter)	Iterative 6i/16s
Definition of tumour volume (cut-off)	TBR > 1.8	TBR > 1.6
ROI for TAC definition	90% isocontour in different slices	TBR > 1.6 in slice with FET maximum

### Definition of tumour ROI

**Fig. 3:**
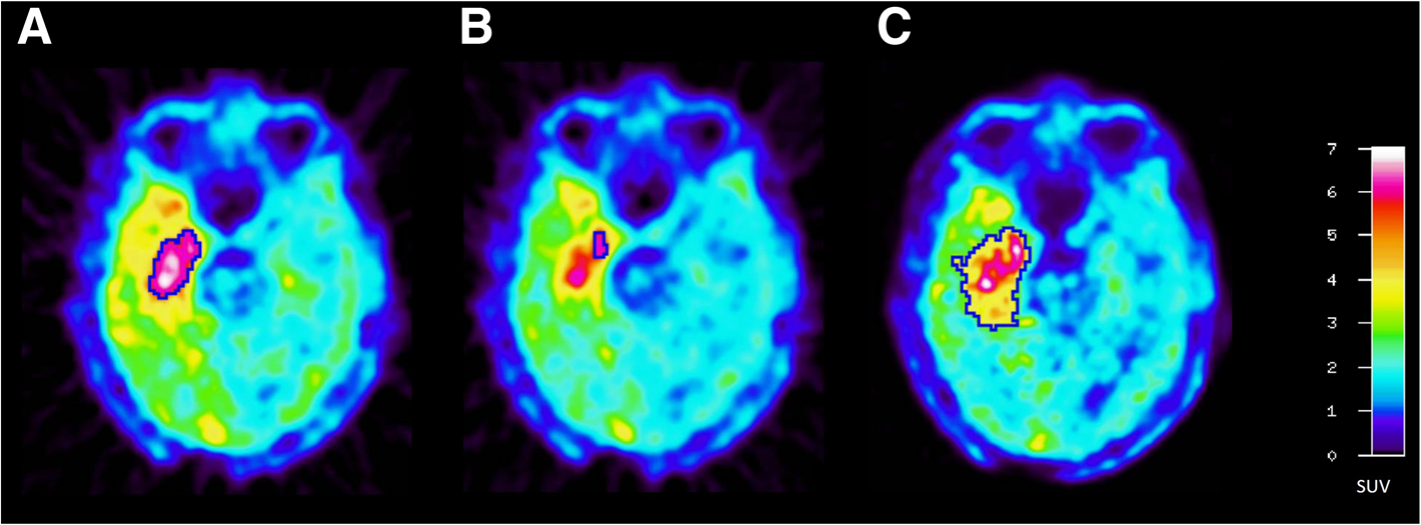
ROI definition in a patient with a glioblastoma in the right temporal lobe. **a** The tumour volume as delineated by TBR > 1.8 based on data reconstruction of centre **A**. **b** The 90% isocontour of the 10–30-min image for TAC generation in centre **A** and **c** the tumour volume as delineated by a TBR > 1.6 in centre B which is also used TAC generation

Further differences between the two centres were identified for the definition of the region of interest (ROI) for the tumour which was used to determine the tumour volume (T_vol_) and the TBR_max_ and TBR_mean_. For the two data sets generated by the different data processing in centres A and B, the definition of these tumour ROIs was based on the summed images of ^18^F-FET uptake from 20 to 40 min after injection in the slice with the maximal ^18^F-FET uptake in the tumour. The background ROI was placed in the contralateral hemisphere to the tumour in an area of normal-appearing brain tissue including white and grey matter, identically for centres A and B. For the data set of centre A, the tumour volume (T_vol_) was delineated using a cut-off of the TBR > 1.8 which is based on the clinical experience in that centre [16, 20]. For the data set of centre B, T_vol_ was delineated using a cut-off of the TBR > 1.6 based on a previous biopsy controlled study [8]. TBR_max_ and TBR_mean_ were calculated by dividing the mean and maximum SUV in the tumour ROI by the mean SUV of the background region in the normal brain tissue. An example is shown in Fig. 3.

For analysis of dynamic PET data, all TACs were corrected for radioactive decay of ^18^F. Centre B used a single tumour ROI which was placed in the slice with maximal tracer uptake in the 20–40-min summation images and which was defined by the cut-off TBR > 1.6 as described above. In centre A, multiple slices of the tumour area were screened and a 90% isocontour ROI of the local tumour maxima was created in the individual slices of the summation image 10–30 min p.i. and TACs were recorded for each slice of the tumour region (Fig. 3b). The TAC with the most negative slope was identified, and the most negative slope being present in at least two adjacent slices was selected for further TAC evaluation. In order to compare the TACs produced by the two different centres quantitatively, the time-to-peak (TTP; time in minutes from the beginning of the dynamic acquisition up to the maximum SUV of the lesion) was determined based on different ROIs determined for centres A and B. Furthermore, we quantified the slope of the TAC in the late phase of ^18^F-FET uptake by fitting a linear regression line to the late phase of the curve (10–40 min p.i.). The slope was expressed in change of SUV per hour.

### Statistical analysis

Descriptive statistics are provided as mean and standard deviation. To compare the results obtained with the different approaches in the two centres based on the same raw data, the paired *t* test and Pearson’s correlation coefficient was used. The signed rank test was used when variables were not distributed normally. *P* < 0.05 was considered significant. The diagnostic performance of the different parameters to differentiate between LGG (WHO grade II) and HGG (WHO grade III and IV) was assessed by analyses of receiver operating characteristic (ROC) curves using the histological confirmation as reference. The area under the ROC curve (AUC) was used to compare the results obtained with different methodologies. Statistical analysis was performed using SigmaPlot software (SigmaPlot v11.0, Systat Software). No correction for multiple testing was included.

## Results

### Static PET data

**Fig. 4:**
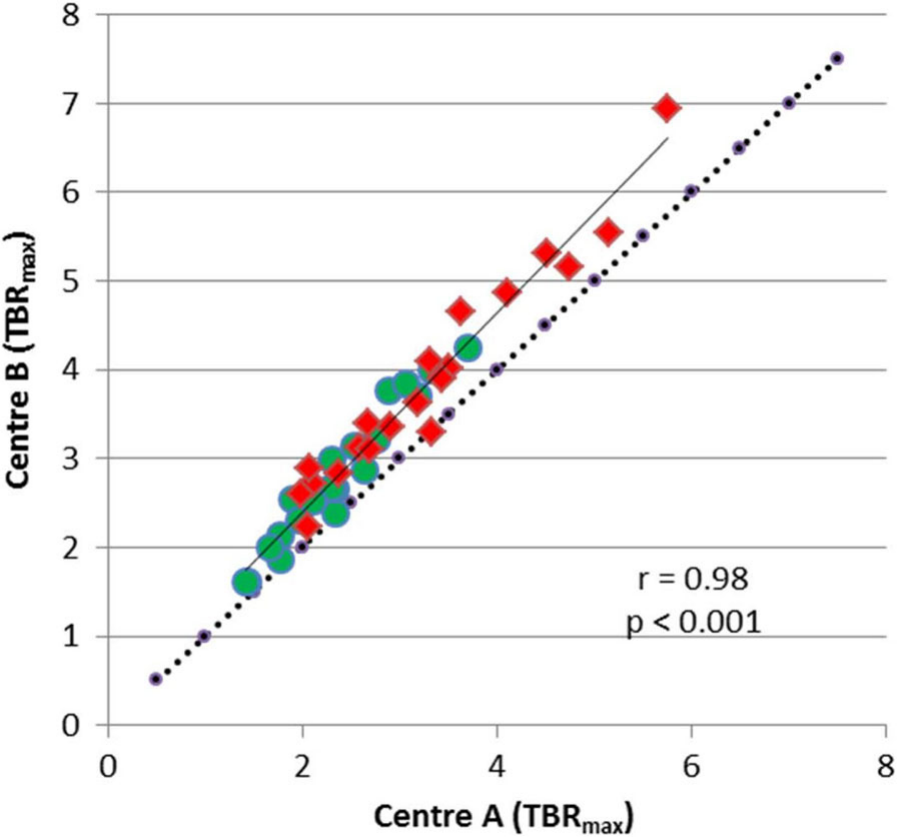
Correlation of TBR_max_ from centres **A** and **B**. There is a significant correlation but a deviation from line of unity indicating higher TBR_max_ values for centre **B**. High-grade tumours are indicated by red symbols and low-grade tumour by green symbols

TBR_max_ values as determined by the different methodologies of centres A and B showed a significant correlation (*r* = 0.98, *p* < 0.001), but there was a deviation from the line of unity towards higher TBR_max_ values for centre B (Fig. 4). The mean values of TBR_max_ in centre A were significantly lower than that in centre B (A/TBR_max_ 2.84 ± 0.99 versus B/TBR_max_ 3.34 ± 1.13, *p* < 0.001) (Table 2). This is also reflected by the lower peak values in the profile of count rates across the tubes with a diameter of 9.3 mm in the Jaszczak phantom (Fig. 2).

**Table 2:** Comparison of ^18^F-FET parameters in centre A and centre B based on the same data set

	Centre A	Centre B	*p* value
Comparison of mean values of FET PET parameters			
TBR_max_	2.84 ± 0.99	3.34 ± 1.13	< 0.001
TBR_mean_	2.20 ± 0.41	2.16 ± 0.41	< 0.04
T_vol_ (ml)	1.14 ± 1.28	1.51 ± 1.44	< 0.001
TTP (min)	22.4 ± 8.3	30.8 ± 6.3	< 0.001
Slope (SUV/h)	−0.06 ± 0.92	0.73 ± 0.69	< 0.001
Comparison of accuracy in differentiating HG and LG gliomas (AUC in ROC analysis)			
TBR_max_	0.77	0.78	n.s.
TBR_mean_	0.78	0.76	n.s.
T_vol_	0.78	0.76	n.s.
TTP	0.73	0.78	n.s.
Slope	0.76	0.72	n.s.

n.s. = not significant

ROC analysis yielded a similar AUC for the differentiation of high-grade and low-grade gliomas using the TBR_max_ values determined with the different approaches (AUC TBR_max_ centre A = 0.77 and AUC TBR_max_ centre B = 0.78) (Table 2).

**Fig. 5:**
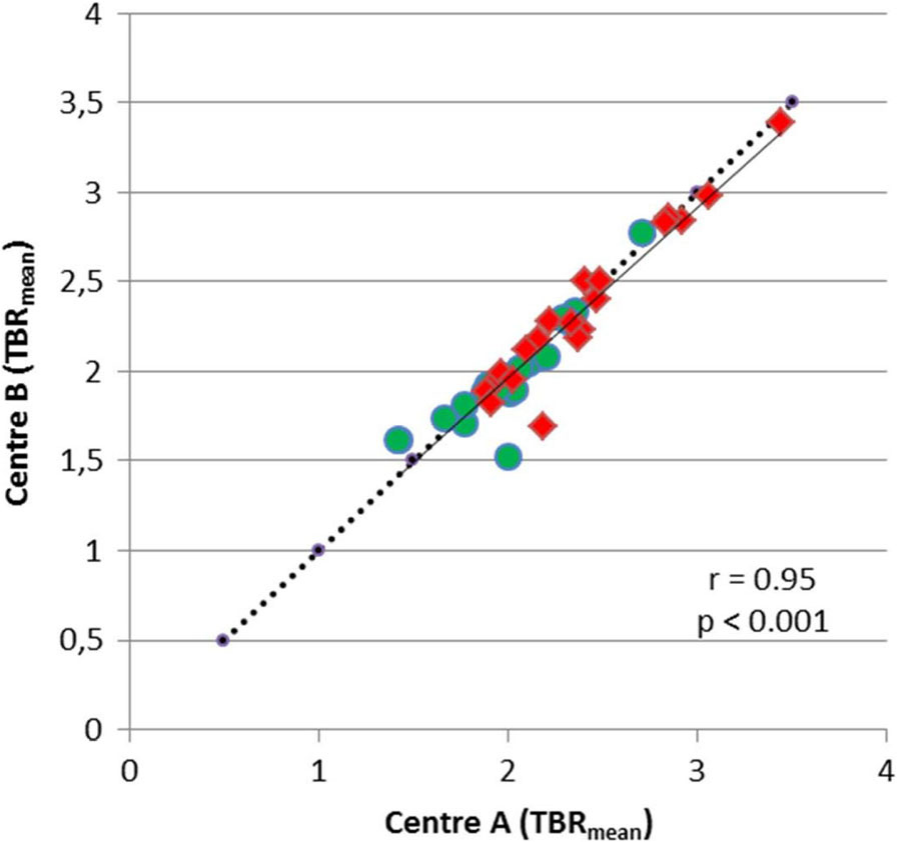
Correlation of TBR_mean_ from centres **A** and **B**. There is a significant correlation which is close to the line of unity. High-grade tumours are indicated by red symbols and low-grade tumour by green symbols

TBR_mean_ values as determined by the different methodologies of centres A and B showed a significant correlation (*r* = 0.95, *p* < 0.001), which was nearly congruent with the line of unity (Fig. 5). The mean values of TBR_mean_ were nearly identical although a weak significance was noted in the paired *t* test (A/TBR_mean_ 2.20 ± 0.41 versus B/TBR_mean_ 2.16 ± 0.41, *p* < 0.04) (Table 2). ROC analysis yielded a similar AUC for the differentiation of high-grade and low-grade gliomas using the TBR_mean_ values determined with the different approaches (AUC TBR_mean_ centre A = 0.78 and AUC TBR_mean_ centre B = 0.76) (Table 2).

T_vol_ values calculated according to the different methodologies of centres A and centre B showed a significant correlation (*r* = 0.99, *p* < 0.001), but there was a significant difference of the mean values (T_vol A_ 1.14 ml ± 1.28 ml versus T_vol B_ 1.51 ml ± 1.43 ml), which leads to relevant differences in the definition of the “biological tumour volume”.

### Dynamic PET data

TTP values as determined by the different methodologies of centres A and B showed a significant correlation (*r* = 0.61, *p* < 0.001), but the correlation coefficient was considerably smaller than for the parameters mentioned above. There was a significant difference of the mean values (TTP A 22.4 ± 8.3 min versus TTP B 30.8 ± 6.3 min, *p* < 0.001).

**Fig. 6:**
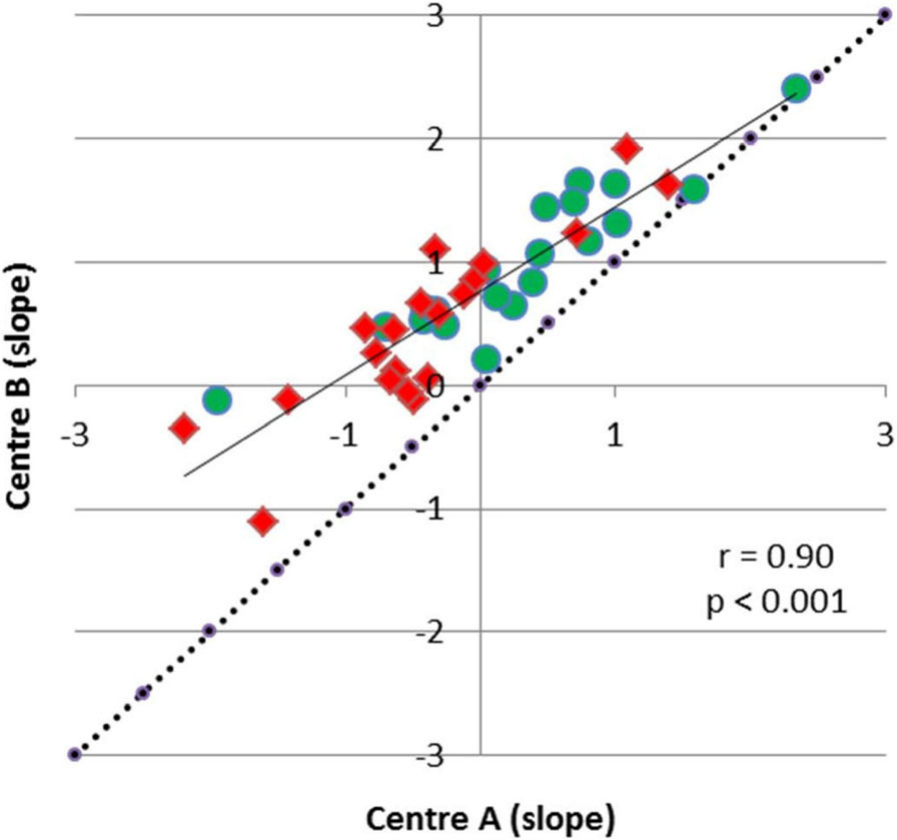
Correlation of slope of the TAC of ^18^F-FET uptake from 10 to 40 min p.i. There is a significant correlation but a deviation from line of unity indicating lower slope values for centre A. High-grade tumours are indicated by red symbols and low-grade tumour by green symbols

**Fig. 7:**
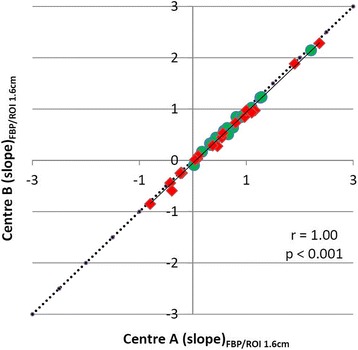
Correlation of slope of the TAC of ^18^F-FET uptake from 10 to 40 min p.i., comparing the effect of different framing in centres A and B. All data were reconstructed by FBP according to centre A and evaluated with a circular ROI with fixed a diameter of 1.6 cm. There is a highly significant correlation which virtually excludes an influence of different data framing. High-grade tumours are indicated by red symbols and low-grade tumour by green symbols

Slope values calculated according to methodology of centres A and B showed a significant correlation (*r* = 0.90, *p* < 0.001) (Fig. 6). There was a significant difference of the mean values (slope A −0.06 ± 0.92 SUV/h versus slope B 0.73 ± 0.69 SUV/h). For both TTP and slope, ROC analysis yielded a similar AUC for the differentiation between HGG and LGG determined with the two different approaches (Table 2). Comparison of the slope values in both centres with framing as the only variable using the same reconstruction method (FBP) and ROI size (Ø 1.6 cm) for both data sets showed a correlation close to the line of unity (Fig. 7). Thus, an influence of different data framing in centres A and B could be excluded.

## Discussion

PET using radiolabelled amino acids is gaining increasing interest for the diagnostics of brain tumours because conventional MRI is limited in differentiating tumour tissue from nonspecific tissue changes, especially after therapy [21]. Recently, the Response Assessment in Neuro-Oncology (RANO) working group—an international effort to develop new standardized response criteria for clinical trials in brain tumours—has recommended the additional use of amino acid PET imaging for brain tumour management [5]. The longest established amino acid tracer ^11^C-MET has been replaced in many neuro-oncology centres by the more convenient ^18^F-FET, and several thousand ^18^F-FET PET scans have been performed in some centres in recent years [22]. The broad clinical use of ^18^F-FET PET requires comparable quantitative parameters, but as yet, the reported cut-off values of different parameters like TBR_mean_, TBR_max_, TTP, slope and T_vol_ for tumour grading or differentiation of recurrent tumour from treatment related changes appear to vary among different centres. It remains unclear whether diverging results are caused by the composition of the study population, differences in the technical equipment or differences in data processing or a combination thereof.

In this study, we have identified methodological differences between two centres in Germany which have a high frequency of ^18^F-FET PET investigations and contributed to the clinical evaluation of ^18^F-FET PET through numerous publications (for review, see [3, 5]). Since in both centres, the previous publications on ^18^F-FET PET studies were performed with the same PET scanner, differences in the technical equipment between the centres can be excluded. A comparison of data processing, however, identified methodological differences between the centres concerning the framing of PET data, data reconstruction, tumour delineation and ROI definition to evaluate tracer kinetics in the tumour. The effects of these methodological differences on quantitative parameters of FET PET were examined in a collective of 40 brain tumour patients.

One frequently used parameter for brain tumour characterization is the TBR_max_ which showed approximately 15% lower values when generated with the methodology of centre A than with that of centre B. In agreement with these findings, we observed lower peak values in a phantom study when data were reconstructed according to the method of centre A. In accordance with these findings, the reported average TBR_max_ values in primary brain tumours are lower in centre A than in centre B (HGG: A/TBR_max_ 3.3 ± 1.2 versus B/TBR_max_ 3.6 ± 1.4, LGG: A/TBR_max_ 2.1 ± 1.0 versus B/TBR_max_ 2.4 ± 1.0) [23, 24]. Nevertheless, the cut-off values of the TBR_max_ for differentiating between HGG and LGG in those studies were similar (cut-off: A/TBR_max_ 2.7, B/TBR_max_ 2.5), and the accuracy of differentiating between HGG and LGG based on the TBR_max_ appears to be similar to both centres. For the differentiation of recurrent tumours from treatment-related changes which is a frequent clinical question, the reported cut-off value for TBR_max_ was 2.0 in centre A and 2.3 in centre B which might also reflect the difference in data processing in the two centres [12, 25]. TBR_mean_ values generated with the methodology of centres A and B showed a small but significant difference in the paired *t* test (A/TBR_mean_ 2.20 ± 0.41 versus and B/TBR_mean_ 2.16 ± 0.41, *p* < 0.04), but this difference would no longer be significant if a correction for multiple testing were applied.

A major difference between both centres was observed for the definition of the biological tumour volume. The approach of centre A led to approximately 25% smaller tumour volumes than that of centre B which is of substantial importance since ^18^F-FET PET is increasingly used when planning therapeutic interventions such as surgery and radiotherapy. It has to be considered that the approach of centre A is based on clinical experience while the approach of centre B was developed on the basis of a biopsy-controlled study which from a scientific point of view has to be regarded as more reliable [8]. On the other hand, that study is based on 52 biopsies in 31 patients only, which appears not sufficient to establish a clinical standard. Furthermore, only patients with newly diagnosed brain tumours were included, and the optimal threshold might be different for pretreated patients who can present slight unspecific tracer uptake at the primary tumour site. Therefore, further biopsy-controlled studies are needed to clarify this clinically important aspect.

The evaluation of dynamic ^18^F-FET PET by the approach of centre A yielded shorter TTP values and lower values for the slope in the late phase of the TAC. A detailed analysis showed that the different framing procedures in centres A and B did not influence the dynamic parameters (Fig. 7) and also the ROI definition by 90% isocontour instead of TBR > 1.6 caused only minor differences. The decisive difference was caused by the more extensive search for tumour areas with negative slope in the entire tumour volume in centre A. This approach appears to be advantageous but is technically more challenging. On the other hand, we did not detect a significant difference between the two approaches for the differentiation between HGG and LGG by ROC analysis in this group of patients.

An important reason for the differences in TBR_max_ and T_vol_ observed in both centres is the different spatial resolution of the PET scans which is caused by the different reconstruction methods. It appears that the use of FBP in centre A is a major cause of the observed differences which is also evident from the phantom study (Fig. 2). This observation is in line with previous studies comparing the difference of FBP to OSEM on quantification of glucose metabolism which all report higher SUVs for OSEM as compared to FBP [26–28]. Since in newer PET systems, data processing is generally based on iterative reconstruction differences between centres which may become smaller in the future. In any case, the spatial resolution of PET scans should be comparable when applying threshold values for the definition of ROIs in the tumour area with FET PET. It is therefore necessary to adapt the reconstruction parameters in order to achieve a similar spatial resolution in various PET systems. Kinetic parameters are less strongly influenced by spatial resolution but are altered by the selection of specific tumour regions.

It needs to be considered that owing to the lack of molecular data for histological analysis, tumour classification was based on the WHO classification from 2007 instead of the new one from 2016, which includes molecular parameters [18, 29]. Therefore, the AUC values obtained for tumour grading in this study might not be directly applicable to the current classification, and an influence on the comparison of the AUC values for centres A and B cannot be excluded.

## Conclusions

The differences in data processing between the two centres lead to considerable differences especially for TBR_max_, slope, TTP and T_vol_. Although there was a strong linear correlation between TBR_max_ and T_vol_ values of both centres, the absolute values cannot directly be compared. Absolute TBR_mean_ values, by contrast, seem comparable between both centres. However, concerning the evaluation of dynamic ^18^F-FET PET, both centres achieved comparable high accuracy to discriminate between HGG and LGG. A standardization of data processing and protocols for ^18^F-FET-PET is needed in order to make clinical results comparable.
